# Smoking in Hospital

**Published:** 1917-09-29

**Authors:** 


*To the Editor of* The Hospital.

### Smoking in Hospital.

*To the Editor of* The Hospital.

Sir,—May I be allowed a little space in your paper to air my views on the answers in The Hospital of September 15, 1917, to the question regarding smoking in hospital?

"Sister" in the article seems to lose sight of the fact that a matron to her nurses ought to stand for truth. Consequently the attitude looked for in a matron is an honest one. If a matron indulges in a cigarette in the privacy of her sitting-room as a relaxation, how can she fairly condemn the same habit in any of her nursing staff in their off-duty time? To say that because a nurse smokes she must necessarily smell of stale tobacco is nonsense; and as for her hands being stained with nicotine, the nurse does too much scrubbing for her hands to remain long stained with anything. And in any case, if she cannot hold a cigarette properly, she can buy a holder for twopence. As smoking on duty is obviously impracticable, no nurse with any sense of the fitness of things would attempt such a proceeding on the sly, any more than she would indulge in any other form of relaxation on the sly. Smoking in moderation cannot be demoralising; one thought of our boys in France should convince "Sister" of this fact. "Sister" has evidently not known what it means to be training in a big city far from home, and with no friends' houses to visit, let alone smoke in. Why should a nurse not smoke in uniform, *off* duty, when our gallant young residents smoke in their hospital coats *on* duty? I do agree with "Sister," where smoking is recognised it should not be indulged in in a common sitting-room, to the possible annoyance of non-smokers.

"Timeo Incendium's" objection to smoking on account of risk of fire is the best reason set down against smoking in hospital. Her objection on the ground that the nurse who can't afford to smoke is led away by the nurse who can, is far-fetched. Nowhere else so much as in hospital are women so ready to admit that they are hard up, as every nurse knows. And only the snobs (unfortunately some are to be found in the profession) would be so stupid as to make pretences in this direction. The suggestion that nurses should sacrifice all their smokes to the Tommies is absurd. We all, I hope, make some sacrifices for our boys in France, but one woman is not in a position to arbitrate what any other woman's particular sacrifice should be in regard to her soldier lover.

Zaza.

